# Electromyography Exposes Heterogeneity in Muscle Co-Contraction following Stroke

**DOI:** 10.3389/fneur.2017.00699

**Published:** 2017-12-22

**Authors:** Caitlin L. Banks, Helen J. Huang, Virginia L. Little, Carolynn Patten

**Affiliations:** ^1^Neural Control of Movement Lab, Malcom Randall VA Medical Center, Gainesville, FL, United States; ^2^Rehabilitation Science Doctoral Program, University of Florida, Gainesville, FL, United States; ^3^Department of Mechanical and Aerospace Engineering, University of Central Florida, Orlando, FL, United States; ^4^Department of Physical Therapy, University of Florida, Gainesville, FL, United States

**Keywords:** stroke, co-contraction, electromyography, walking, methodology, motor disorders

## Abstract

Walking after stroke is often described as requiring excessive muscle co-contraction, yet, evidence that co-contraction is a ubiquitous motor control strategy for this population remains inconclusive. Co-contraction, the simultaneous activation of agonist and antagonist muscles, can be assessed with electromyography (EMG) but is often described qualitatively. Here, our goal is to determine if co-contraction is associated with gait impairments following stroke. Fifteen individuals with chronic stroke and nine healthy controls walked on an instrumented treadmill at self-selected speed. Surface EMGs were collected from the medial gastrocnemius (MG), soleus (SOL), and tibialis anterior (TA) of each leg. EMG envelope amplitudes were assessed in three ways: (1) no normalization, (2) normalization to the maximum value across the gait cycle, or (3) normalization to maximal M-wave. Three co-contraction indices were calculated across each agonist/antagonist muscle pair (MG/TA and SOL/TA) to assess the effect of using various metrics to quantify co-contraction. Two factor ANOVAs were used to compare effects of group and normalization for each metric. Co-contraction during the terminal stance (TSt) phase of gait is not different between healthy controls and the paretic leg of individuals post-stroke, regardless of the metric used to quantify co-contraction. Interestingly, co-contraction was similar between M-max and non-normalized EMG; however, normalization does not impact the ability to resolve group differences. While a modest correlation is revealed between the amount of TSt co-contraction and walking speed, the relationship is not sufficiently strong to motivate further exploration of a causal link between co-contraction and walking function after stroke. Co-contraction does not appear to be a common strategy employed by individuals after stroke. We recommend exploration of alternative EMG analysis approaches in an effort to learn more about the causal mechanisms of gait impairment following stroke.

## Introduction

After a stroke, most individuals experience lifelong walking impairments, including forward propulsion deficits, which contribute to metabolically inefficient gait (1–4). Abnormal muscle activation patterns, especially excessive co-contraction, are commonly argued to be a major contributing factor to these walking impairments (5–7). Co-contraction refers to simultaneous activity in agonist and antagonist muscles across the same joint (6, 8). This phenomenon is sometimes called agonist/antagonist co-activation or simply co-activation. However, co-activation can also refer to simultaneous activity in synergist muscles. Here, we will use the term co-contraction and address the relationship between agonist and antagonist muscle co-activity.

Co-contraction is a normal motor control strategy observed in healthy individuals during functional motor tasks. Its presence varies in response to environmental and task demands on different time scales. For example, when encountering uncertainty, such as challenges to posture and balance, increased co-contraction can be observed as an early response to the novel environment (9). Evidence suggests that co-contraction facilitates rapid torque development (10), compensates for non-linearities in muscle properties and torque scaling (11), and counteracts agonist torques and off-axis torques (12, 13). Thus, co-contraction affords a robust mechanism to rapidly counteract perturbations. Co-contraction is also often present when motor tasks are novel or require limb stabilization for performance accuracy. Over a longer time scale (e.g., minutes, hours or days), co-contraction decreases progressively. Indeed, an asymptote in co-contraction often serves as a hallmark of motor learning and adaptation (14, 15). Important to its role as a normal motor control strategy, co-contraction is actively modulated during movements such as walking, occurring more prominently at predictable points in the gait cycle to stabilize joints and enable efficient walking (8).

In contrast to this normal pattern of co-contraction, chronic presence of excessive or invariant co-contraction should reflect pathology (16). Disturbed voluntary muscle activation following stroke is thought to involve excessive co-contraction, which may stem from diffuse descending motor drive or exaggerated stretch reflexes generated in the antagonist muscle by movement (i.e., antagonist restraint) (17, 18). Clinical perspectives have emphasized the presence of excessive and invariant co-contraction in neuropathologic conditions, fueling an expectation that it is ubiquitously present (16, 19–21). However, the literature to date remains inconclusive regarding either its presence or causal role in motor dysfunction (10, 19–21). Furthermore, it is thought that co-contraction contributes to slow, inefficient walking after stroke (5, 6, 22, 23). Importantly, if excessive co-contraction is present during walking after stroke, it should be quantifiable and associated with robust measures of gait impairment, particularly, generation of plantarflexor power.

While objective, quantifiable methods exist for analyzing co-contraction in healthy individuals, the literature provides no “best” method to account for co-contraction as a feature of pathologic muscle activation patterns (24). We surveyed the existent literature and selected two popular metrics. The first, developed by Falconer and Winter, is a metric based on normal gait patterns from ten healthy adults (8). This method computes a ratio of antagonist to agonist electromyography (EMG) activity within each phase of the gait cycle. Another common method quantifies the “wasted contraction” (WC) shared between an agonist and antagonist muscle by denoting the smaller of the two traces as the WC and the remaining EMG activity as the “effective contraction,” which generates movement. This approach was developed by researchers studying upper limb motor adaptation and computational motor control (25). To our knowledge, the WC measure has not been applied to evaluate co-contraction during walking. Both metrics evaluate muscle activation patterns in a manner that does not account for the biomechanical role of each muscle within the task. That is, the larger magnitude EMG signal is assumed to arise from the agonist and the smaller signal from the antagonist. However, this assumption does not always hold, especially after stroke. Impaired EMG amplitude and phasing following stroke may, therefore, require a metric that is sensitive to these changes in muscle roles throughout the gait cycle. EMG normalization also varies considerably when quantifying co-contraction, which may further influence data interpretation and outcomes.

The goal of the present study is to assess the relationship between ankle co-contraction and gait impairment following stroke. Given the challenges involved with detecting co-contraction in a pathologic population, we will investigate the effect of various methods for quantifying co-contraction. Here, we introduce a modified version of the Falconer and Winter metric in which the agonist and antagonist muscle roles are prescribed within each phase of the gait cycle. We will investigate the effects of co-contraction metric and EMG normalization in order to comprehensively assess the presence and magnitude of pathologic co-contraction. Our results will allow us to determine whether, and how, the metric impacts the ability to detect pathologic co-contraction patterns after stroke, while accounting for inconsistencies in the literature that may underlie detection of this phenomenon.

## Materials and Methods

### Subjects

This is a subgroup analysis from a larger study. We included 15 individuals post-stroke and 9 healthy controls. Demographic data are presented in Table 1. Overall, participants were included if they were: greater than 18 years of age, able to walk independently for a distance of at least 15 m with or without an assistive device, and medically stable. Participants post-stroke were included following clinical presentation of a single, unilateral stroke—confirmed by neuroimaging—at least 6 months prior to enrollment. Individuals with brainstem or cerebellar involvement, bilateral involvement, major neurologic or neurodegenerative conditions other than stroke, orthopedic or cardiovascular conditions that precluded walking on a treadmill, or pregnancy were excluded.

**Table 1:** Demographics.

	Control	Stroke
**Demographics**		
*n*	9	15
Sex (m/f)	5/4	14/1
Age (years)	60 ± 9.33	65.87 ± 9.76
Self-selected walking speed (m/s)	1.40 ± 0.2^a^	0.93 ± 0.3^a^
Chronicity (years)		5.52 ± 3.73
Affected side (r/l)		8/7
**Clinical characteristics**		
Routine ankle foot orthosis (AFO) use (*n*)		4
LE Fugl-Meyer Motor Score (/34)		30 (16, 34)
Dynamic Gait Index (/24)		21 (10, 24)
Short Physical Performance Battery (/12)		11 (7, 12)

*Demographic and clinical data are presented mean ± SD and median (range), respectively. Of the four individuals who routinely use an ankle-foot orthosis, three typically use a custom-molded AFO and one uses a prefabricated Aircast^®^*.

*^a^Indicates a significant difference between groups, p < 0.05*.

Testing occurred at the Brain Rehabilitation Research Center in the Malcom Randall VA Medical Center in Gainesville, FL, USA.

### Instrumentation and Protocol

Participants walked on an instrumented split-belt treadmill (Bertec, Columbus, OH, USA, sampling frequency 2,000 Hz) at their self-selected speed. No handrail support was provided and participants wore a modified mountain climbing harness for fall arrest (Robertson Harness, Henderson, NV, USA); no substantial body weight support was provided. Four participants regularly used a custom-molded ankle foot orthosis (*n* = 3) or ankle brace (*n* = 1) on the paretic leg. Two of these participants wore an Aircast^®^ AirSport™ (DJO Global, Vista, CA, USA) for mediolateral support during testing, while all other participants were tested without ankle support. Reflective markers were placed over anatomical landmarks using a modified Helen Hayes marker set (26). Coordinates of the anterior superior iliac spines were located using a digitizing pointer (C-Motion, Inc., Germantown, MD, USA). Locations of the medial and lateral malleoli were digitized for AirCast^®^ wearers due to interference with skin contact. Marker data were recorded by 12 infrared cameras (Vicon MX, Vicon Motion Systems Ltd., Oxford, UK) at 200 Hz.

Surface EMG was recorded using active preamplifiers (MA-420, Motion Lab Systems, Baton Rouge, LA, USA; input impedance >100,000,000 Ω, CMRR >100 dB at 65 Hz, noise <1.2 μV RMS, signal bandwidth 10–2,000 Hz) attached to gel surface electrodes (Cleartrace 2, Conmed, Utica, NY, USA). Skin was abraded and cleaned with alcohol, then electrodes were placed on the muscle belly of the medial gastrocnemius (MG), soleus (SOL), and tibialis anterior (TA) of each leg according to the SENIAM guidelines (27). To assure maximal resolution, amplifier gains were adjusted minimally by visual inspection during isolated ankle movements prior to data collection. EMG data were recorded in Signal (Version 6.0, Cambridge Electronic Design, Cambridge, England) at 2,000 Hz.

Maximal M-waves (M-max) were elicited through stimulation with a Digitimer stimulator (DS-7A or DS-7AH for high-current stimulation, Hertfordshire, UK) using either a hand-held bipolar stimulator probe (CareFusion, Middleton, WI, USA) or a custom monopolar ball electrode. M-max was elicited in the paretic or test leg MG/SOL or TA by supramaximal stimulation of the tibial nerve or common peroneal nerve, respectively (28). M-max amplitudes were averaged across 4–7 consecutive stimulations. One control subject was excluded from M-max analysis because data were overwritten.

Participants were instructed to walk with their arms relaxed at their sides. Data were collected in 1-min walking blocks, with seated or standing rest breaks taken as needed.

### Data Analysis

#### EMG Processing

EMG data were band-pass filtered to remove noise (fourth order Butterworth, cutoff frequency 10–450 Hz), rectified, low-pass filtered (fourth order Butterworth), time-normalized to a 1,001-point gait cycle, and signal averaged (60 cycles), to establish a linear envelope. The ideal low-pass filter frequency was determined by each individual’s mean stride time (cutoff frequency 5/stride time Hz; control 4.6 ± 0.3 Hz, stroke 3.9 ± 0.5 Hz), as recommended by Shiavi et al. (29). The linear envelopes were then amplitude-normalized in each of three ways: (1) no normalization (non), (2) to the maximum value across the gait cycle (max), or (3) to M-max. Data were processed using custom MATLAB scripts (The MathWorks r2015a, Natick, MA, USA).

#### Quantification of Co-Contraction

The gait cycle was divided into seven bins using gait events extracted from ground reaction force and heel marker data. The events defined bins following the standard established by the Rancho Los Amigos Medical Center Pathokinesiology Lab: loading response (LR), mid-stance (MSt), terminal stance (TSt), pre-swing (PSw), initial swing (ISw), mid-swing (MSw), and terminal swing (TSw) (30). The three swing bins each represent one-third of the swing phase.

**Figure 1:**
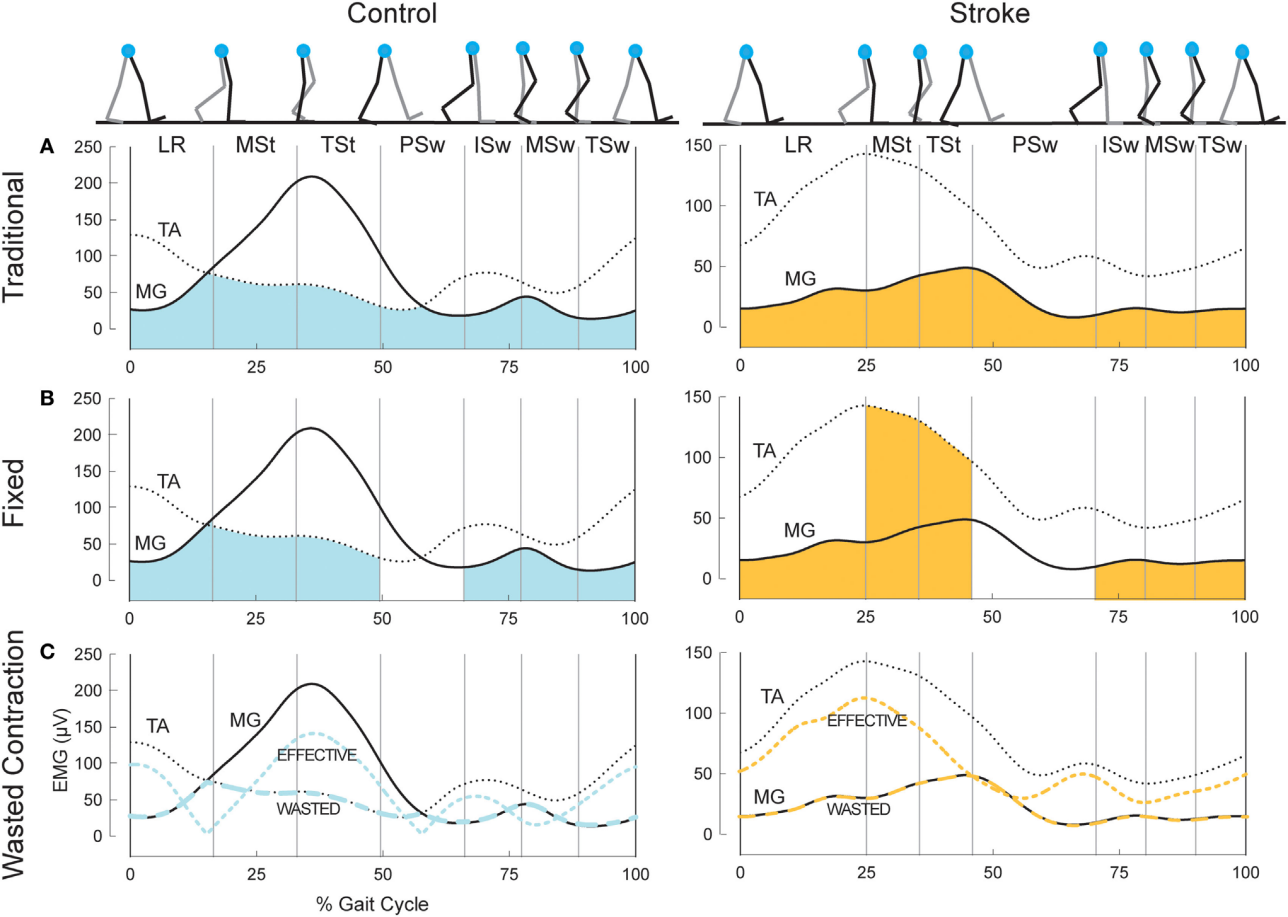
Example co-contraction metrics. **(A)** Eight gait events dividing the gait cycle into seven phases: loading response (LR), mid-stance (MSt), terminal stance (TSt), pre-swing (PSw), initial swing (ISw), mid-swing (MSw), and terminal swing (TSw). Illustrated immediately below, is example EMG in the medial gastrocnemius (MG, solid) and tibialis anterior (TA, dotted) for a healthy control (left) and an individual post-stroke (right). The shaded area reflects the area under the lower trace at any point, used as the antagonist in the traditional co-contraction index calculations. **(B)** Illustration using the same example EMG as above, however the shaded area reflects the area under the muscle that should be the antagonist within each phase. PSw is not shaded because the antagonist muscle typically switches between MG and TA in this phase. **(C)** The resulting wasted contraction for the same subjects, the lower of the two traces (wide dashes), and the effective contraction, which is calculated as the higher trace minus the lower trace (narrow dashes). The wasted contraction index is the mean of the wasted contraction, expressed as a percentage of the maximum effective contraction.

We used three metrics to quantify co-contraction. The first metric, which we will denote as CI_traditional_, was first described by Falconer and Winter (8). This metric represents a ratio of agonist/antagonist overlap to total muscle activation. The total antagonist activity within each bin, I_ant_, is calculated as the area under the curve created by the smaller EMG envelope as indicated by the shaded region in the top row of plots in Figure 1. The total activity, I_tot_, is the sum of the agonist and antagonist EMG areas within a given pair of muscles (i.e., MG/TA or SOL/TA). The co-contraction index can then be quantified as:

(1) $${\text{CI}}_{\text{traditional}}=\frac{2{\text{I}}_{\text{ant}}}{{\text{I}}_{\text{tot}}}*100.$$

A CI value of 100% represents total co-contraction, while a value of 0% represents pure agonist activation. This measure has been widely cited and applied in the literature (31–35). We calculated two co-contraction indices for each phase of the gait cycle, one with the MG/TA pairing, and one with the SOL/TA pairing.

The second metric, CI_fixed_, is analogous to the traditional Falconer and Winter metric, except the agonist/antagonist muscle relationship was fixed to the biomechanical function of these muscles within each of the seven bins of the gait cycle, as demonstrated by typical EMG patterns of healthy individuals (Figure 1, middle plots). During LR, ISw, MSw, and TSw, the TA should be the agonist muscle, while the MG should be the antagonist:

(2) $${\text{CI}}_{\text{fixed}}=\frac{2{\text{I}}_{\text{MG}}}{{\text{I}}_{\text{tot}}}*100.$$

During MSt and TSt, the MG should be the agonist and the TA the antagonist muscle:

(3) $${\text{CI}}_{\text{fixed}}=\frac{2I_{\text{TA}}}{I_{\text{tot}}}*100.$$

In health, the ankle muscles switch agonist/antagonist roles in PSw; thus, we were unable to calculate a co-contraction index during PSw using this method. CI_fixed_ for the SOL/TA pairing was calculated in the same manner as Eqs 2 and 3 above; however, the SOL was used as the antagonist muscle during LR and the swing phases.

The final metric, illustrated in the bottom plots of Figure 1, was developed by Thoroughman and Shadmehr to represent the amount of “wasted contraction” that occurs due to co-contraction (25). Similar to CI_traditional_, the smaller of the two EMG envelopes is designated to represent the contraction that is wasted by simultaneous activity in opposing muscles. This amount can be subtracted from the EMG envelope of the larger signal to determine “effective contraction,” or the amount of agonist activation that effectively performed the movement. The wasted and effective contraction traces can be divided by the maximum effective contraction, resulting in units expressed as the percentage of maximum effective contraction. This step was necessary to compare the effects of EMG normalization since the units of wasted and effective contractions are the same as the units of the EMG traces from which they are based (e.g., microvolts). The mean wasted and effective contractions were calculated for each gait phase.

#### Quantification of Motor Impairment

##### Clinical and Functional Assessments

We used two functional assessments of motor impairment: the lower extremity Fugl-Meyer Assessment of Motor Performance (LE FMA) and walking speeds (36). Self-selected and fastest comfortable walking speeds (SSWS and FCWS, respectively) were captured using 3–5 passes on a 16-foot GaitRite pressure-sensitive walkway (Platinum Plus System, Version 3.9, Havertown, PA, USA). SSWS involved walking at a casual, comfortable pace. Fastest comfortable speed was assessed as the fastest speed the participant could safely attain when walking, “as if you are crossing the street and the walk signal changed to a red hand.” All clinical and functional assessments were performed by a licensed physical therapist (VLL).

##### Ankle Power

**Figure 2:**
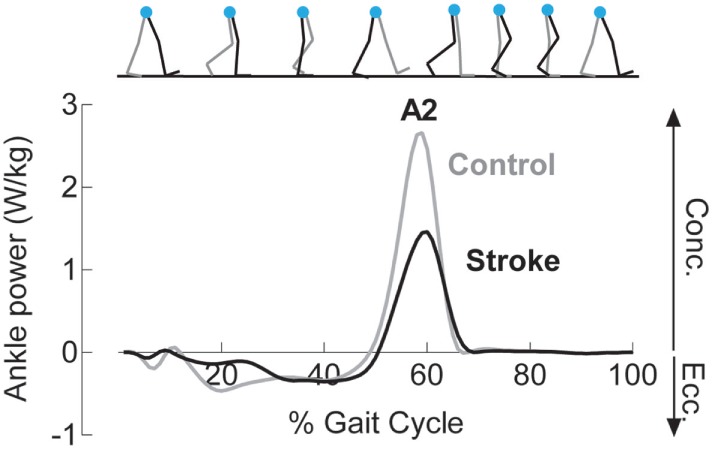
Reduced concentric ankle plantarflexor power is noted following stroke. Example ankle power curves, derived from inverse dynamics, for a healthy control (gray) and an individual following stroke (black) demonstrate diminished peak concentric plantarflexor power (A2) late in the stance phase of gait.

We used ankle power to quantify a key biomechanical aspect of walking function. Power was calculated using inverse dynamics by the following formula:

(4) P=M⋅ω,

where *P* is the rate of work done by the ankle muscles (i.e., power), M is the joint moment, and ω is the angular velocity (37, 38). An example ankle power curve is shown in Figure 2. The second peak of the ankle power curve, or A2, is prominent in late stance for both normal and pathologic gait (37, 39). A2 is often diminished with aging and in individuals post-stroke (39, 40). Although A2 scales with walking speed, the deficit in these individuals is present even when compared to speed-matched controls (41, 42). Because the plantarflexors are the primary mediators of A2 and A2 and accounts for both joint motion and muscular output through torque generation (43), this outcome is highly sensitive and relevant to functional changes after stroke.

### Statistics

Data were tested for normality using a Shapiro–Wilk *W* test. In the cases where the assumption of normality was not met (all cases except SOL/TA CI_traditional_), data were transformed using a base 10 logarithmic transformation. In the final case, raw data were analyzed. Separate two-factor ANOVAs assessed the effects of group (control or stroke) and normalization (maximum value, M-max, or no normalization) on each of the three co-contraction indices and muscle pairs (MG/TA, SOL/TA). Tukey’s Honestly Significant Difference was performed *post hoc* when significant effects were detected. To account for multiple comparisons, significance was established using a Bonferroni corrected value of *p* < 0.0083. Spearman’s correlations assessed the relationship between co-contraction and walking function. The Bonferroni corrected significance level for correlation analyses was *p* < 0.0125. Statistical testing was performed in JMP Pro 11 (SAS Institute, Inc., Cary, NC, USA).

## Results

**Figure 3:**
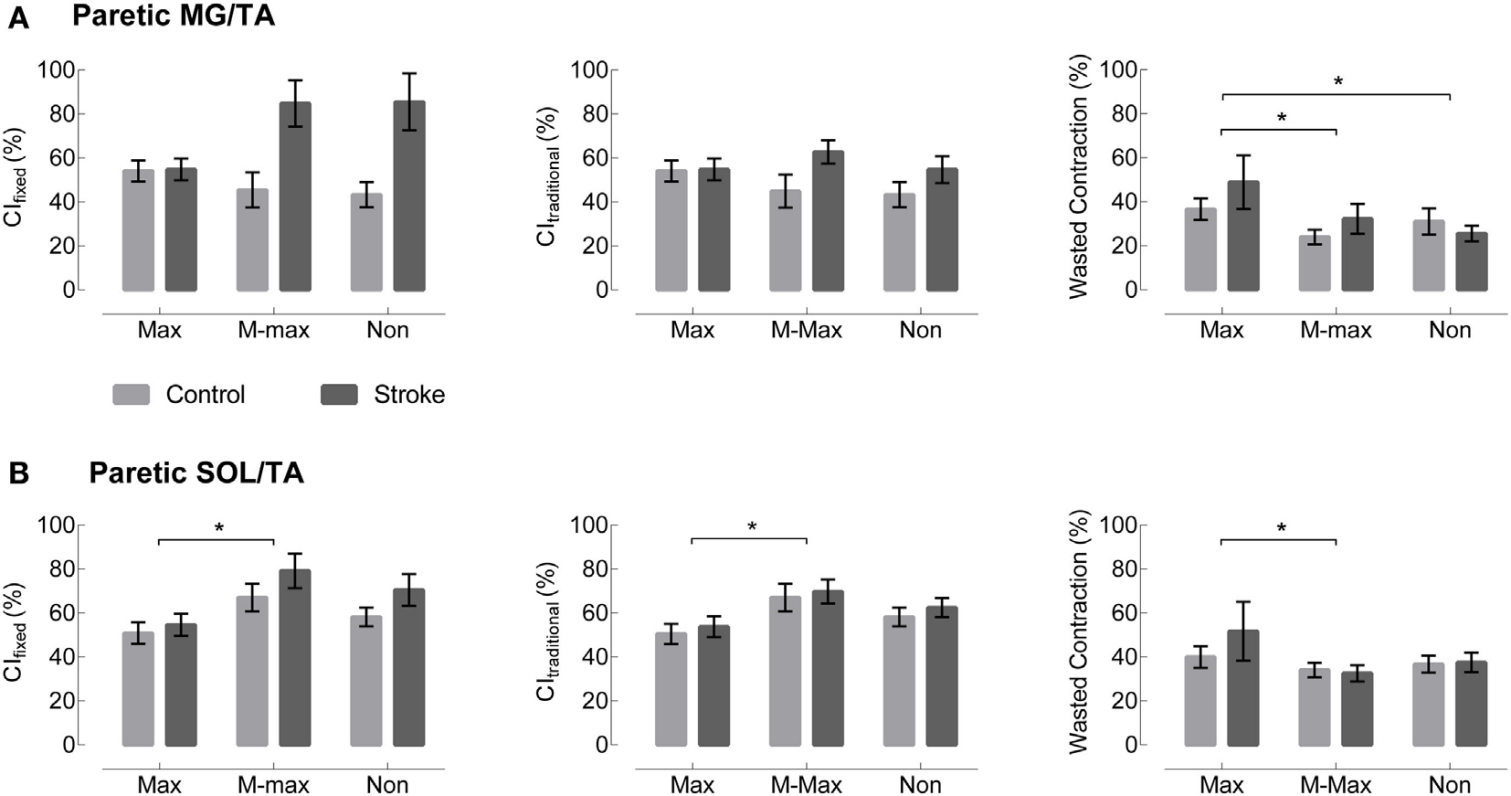
Paretic leg co-contraction following stroke is not different from healthy controls. **(A)** Co-contraction of the medial gastrocnemius and tibialis anterior (MG/TA) in terminal stance is shown by three EMG normalization methods: maximum value (Max), maximal M-wave (M-max), and non-normalized (Non). Plots moving left to right represent three different co-contraction indices: a co-contraction ratio with fixed muscle roles derived from referencing healthy EMG (CI_fixed_, left), a traditional index for co-contraction during normal walking (CI_traditional_, center), and an index of the “wasted contraction” produced by antagonist activation countering agonist activation (right). There are no group differences between stroke (gray) and control (black) in any of the three comparisons. **(B)** Co-contraction of the soleus and tibialis anterior (SOL/TA) in terminal stance. Again, there were no significant differences between control and stroke. *Indicates significance with a Bonferroni-corrected *p* < 0.0083.

**Figure 4:**
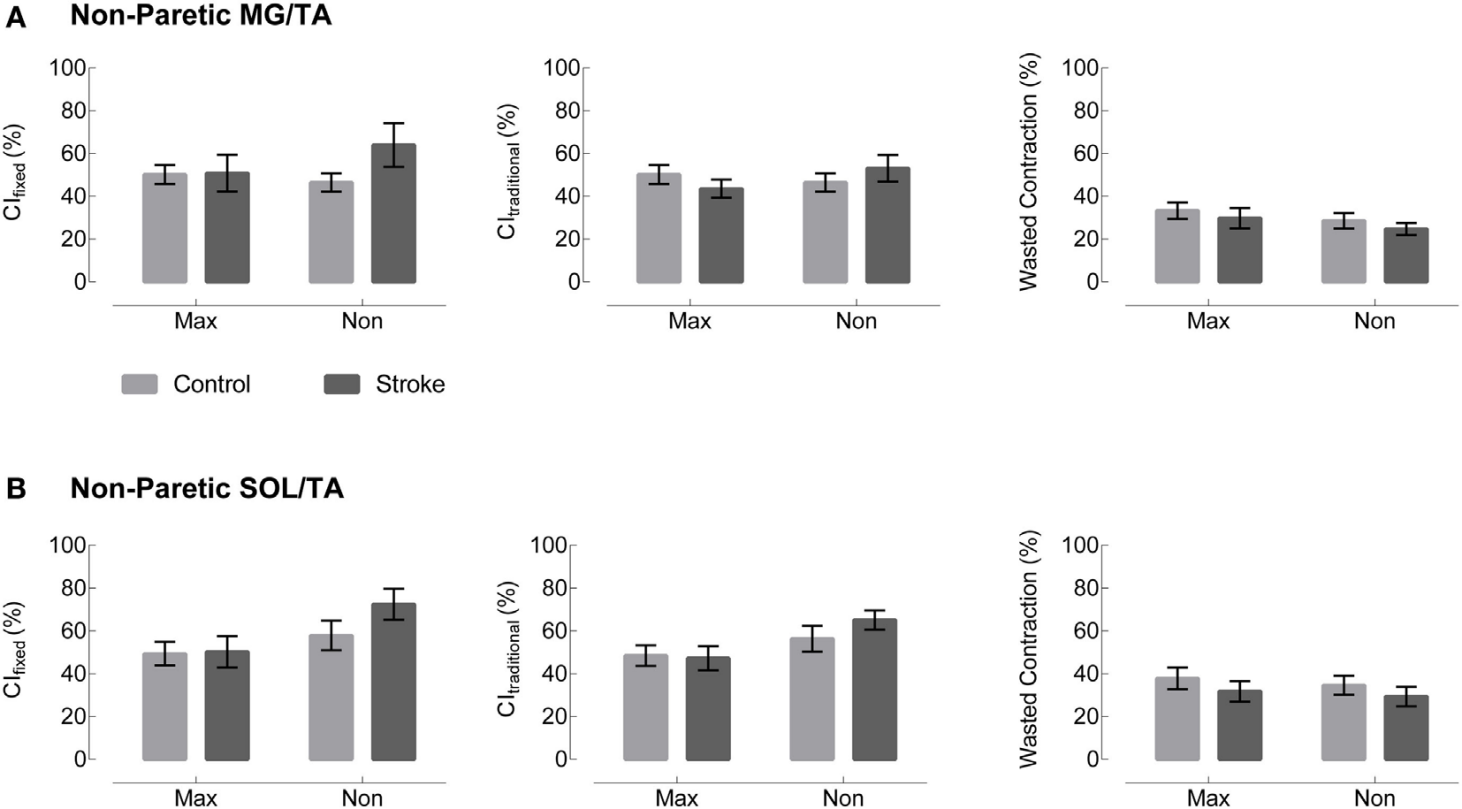
Non-paretic leg co-contraction appears unchanged across group, metric, and normalization method. Plots moving left to right represent three different co-contraction indices: a co-contraction ratio with fixed muscle roles derived from referencing healthy EMG (CI_fixed_, left), a traditional index for co-contraction during normal walking (CI_traditional_, center), and an index of the “wasted contraction” produced by antagonist activation countering agonist activation (right). Co-contraction in terminal stance appears unchanged between control (gray) and stroke (black) in both **(A)** the medial gastrocnemius and tibialis anterior (MG/TA) and **(B)** the soleus and tibialis anterior (SOL/TA).

Visual assessment of EMG patterns during walking revealed consistent patterns in healthy controls, accompanied by vast heterogeneity in the stroke group. Differences between healthy and stroke subjects are most relevant in TSt, where peak plantarflexor EMG tends to occur. TSt is also the gait phase when plantarflexor EMG contributes to A2—the most robust measure of gait function employed in this study. Moving forward, all results will be presented during TSt unless specified otherwise. Group co-contraction responses during TSt can be visualized by metric in Figure 3 for the paretic leg and Figure 4 for the non-paretic leg. Because the core findings are largely similar between legs and we are primarily interested in paretic leg function, we performed statistics only on the paretic leg.

The metric adjusted for the muscles’ biomechanical roles, CI_fixed_, showed no significant main effect of Group or Group × Normalization interaction. In the SOL/TA muscle pairing, there was a significant main effect of normalization method (*p* = 0.0032, Figure 3). *Post hoc* testing revealed that M-max normalization produced greater CI_fixed_ than maximum value normalization (*p* = 0.0024). The MG/TA pairing revealed no significant main effects.

CI_traditional_ resulted in values similar to CI_fixed_ during TSt, and the statistical findings are largely the same. Both muscle pairings revealed no significant main effects of Group or Group × Normalization interactions. The SOL/TA pairing revealed a significant main effect of Normalization (*p* = 0.0051). *Post hoc* testing revealed that M-max normalization was again greater than maximum value normalization (*p* = 0.0035). The MG/TA pairing revealed no significant main effects.

**Figure 5:**
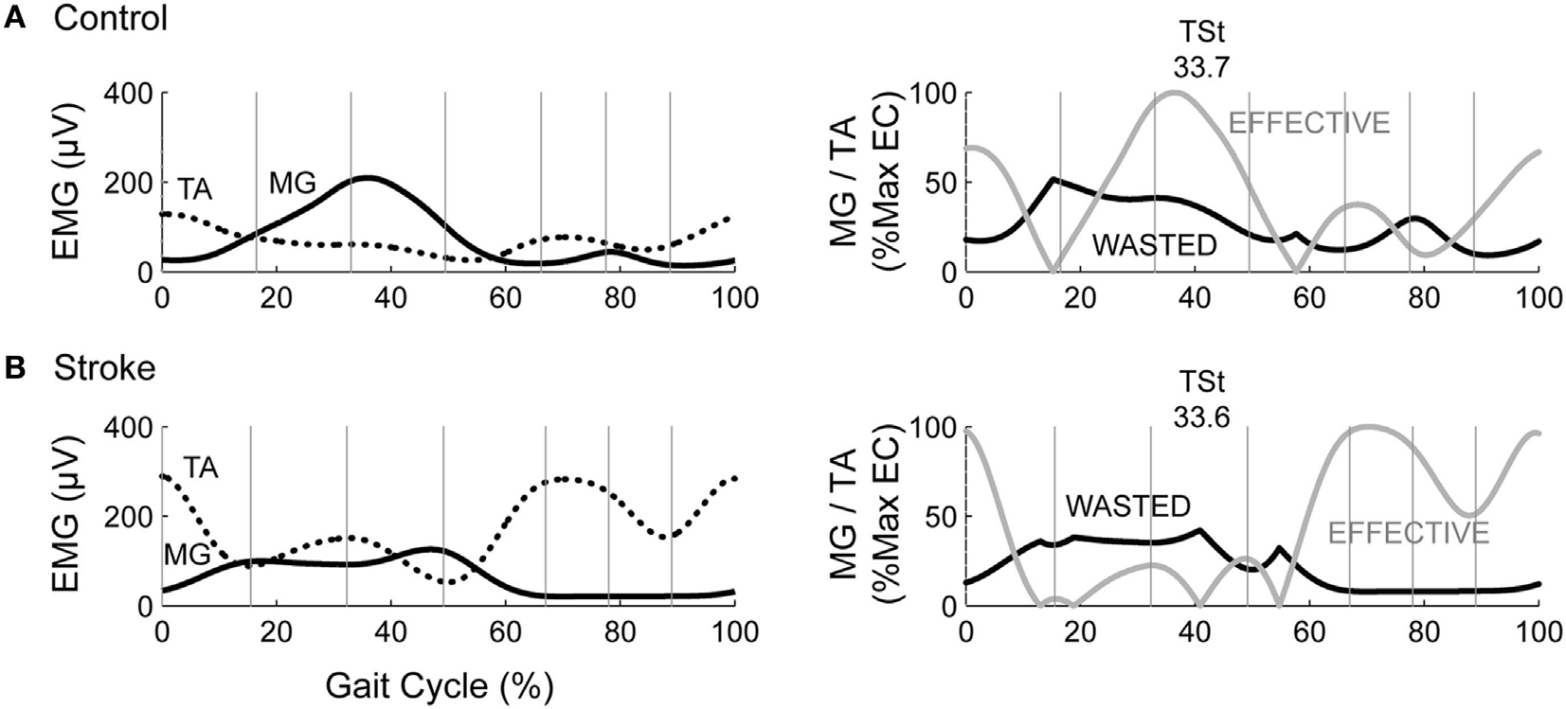
Two subjects show vastly different EMG with same wasted contraction (WC) value in terminal stance. The left column depicts example EMG for the medial gastrocnemius (MG) and tibialis anterior (TA) muscles in **(A)** a healthy control and **(B)**. an individual post-stroke. The right column depicts the resultant wasted and effective contraction, with WC in TSt denoted above each plot.

The WC, when expressed as a percentage of the effective contraction, has the potential to create outliers when the two EMG traces are similar in magnitude. In the case of maximum value normalization, one subject’s EMG produced an extreme value for both the MG/TA and SOL/TA muscle pairings, corresponding to *z*-scores of 6.3 and 7.0, respectively. Since no data transformation could normalize the dataset with outliers of that magnitude, we adjusted each of the outliers to a *z*-score of 3. This step allowed for adequate statistical comparison within the remaining dataset. Both the MG/TA and SOL/TA muscle pairings revealed only a significant main effect of Normalization (*p* = 0.0008 and *p* = 0.0005, respectively). *Post hoc* testing for the MG/TA pairing revealed that maximum value normalization produced greater WC values than both M-max (*p* = 0.0044) and WC derived from non-normalized EMG (*p* = 0.0018). In the SOL/TA muscle pairing, WC was greater with maximum value normalization than M-max normalization (*p* = 0.0004). Because the adjusted outlier would have created an even higher mean value for maximum value-normalized WC, these significant findings are consistent with the original direction of the adjusted outlier. When calculating WC, we observed an interesting phenomenon. Two subjects with visually different EMG patterns revealed comparable WC values in TSt (Figure 5). Aside from the maximum value normalization condition, the WC values tended to have low variability, despite obvious visual differences present in the original EMG. This circumstance creates a problem for interpreting the WC results, which we will revisit in the Section in the discussion.

**Figure 6:**
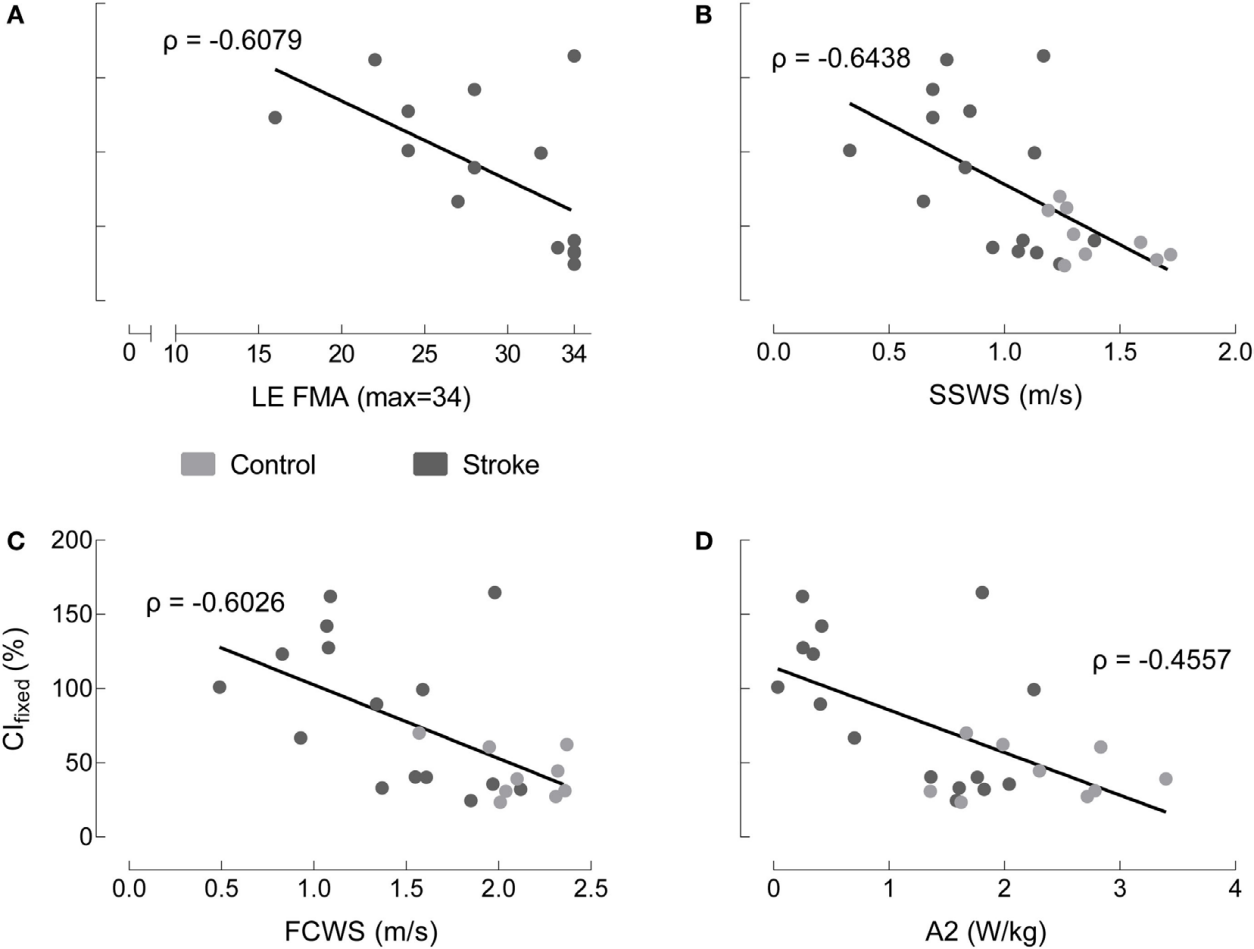
Co-contraction index in terminal stance correlates to some, but not all, indices of motor function. Medial gastrocnemius/tibialis anterior CI_fixed in terminal stance with non-normalized EMG varies as a function of: **(A)** lower extremity Fugl-Meyer (LE FMA), **(B)** self-selected walking speed (SSWS), **(C)** fastest comfortable walking speed (FCWS), and **(D)** peak concentric ankle plantarflexor power (A2) for healthy controls (gray), individuals post-stroke (black). The only correlations reaching statistical significance occur with SSWS and FCWS.

Non-normalized CI_fixed_ values are moderately correlated with functional metrics across groups. We observed similar results across all co-contraction indices. Because non-normalized data are most straightforward to interpret, we chose this metric to perform correlations with functional measures. Therefore, C*I*_fixed_ serves as representative of the patterns present across metrics. Four comparisons were made between CI_fixed_ and LE FMA, SSWS, FCWS, and A2 (Figure 6). CI_fixed_ was not significantly correlated with either LE FMA (Spearman’s ρ = −0.6079, *p* = 0.0162) or A2, our most sensitive assessment of walking function (ρ = −0.4557, *p* = 0.0252). CI_fixed_ was significantly correlated with both SSWS (ρ = −0.6438, *p* = 0.0007) and FCWS (ρ = −0.6026, *p* = 0.0018).

## Discussion

**Figure 7:**
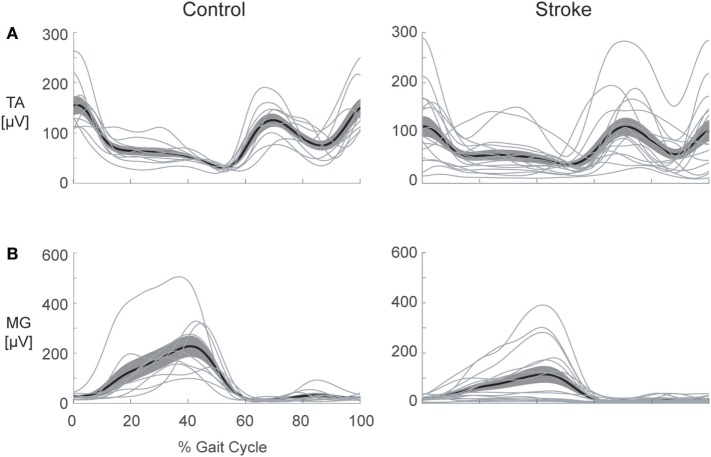
Heterogeneity in electromyography (EMG) activation is present following stroke. Healthy control (left) and stroke (right) EMG envelopes are illustrated for the: **(A)** tibialis anterior (TA) and **(B)** medial gastrocnemius (MG). The heavy line depicts the ensemble average for each group and the cloud illustrates the SEM with individual subject responses overlaid (thin lines). Considerable variation in activation patterns of each muscle is apparent within the stroke group, illustrating the difficulty inherent in selecting a single metric to capture all of the possible deviations from the pattern of healthy controls.

Despite our systematic analysis of co-contraction during gait, we are unable to conclude that co-contraction is ubiquitously present post-stroke. As illustrated in Figure 7, this sample of individuals post-stroke revealed vast heterogeneity in EMG patterns, and no distinct pattern of co-contraction emerged. Furthermore, regardless of the metric used to assess co-contraction, our results fail to indicate clear differences between stroke and healthy controls. Although results reported here are limited to the TSt phase of gait, this finding is consistent across all gait phases. Nearly four decades of literature discuss pathological co-contraction after stroke, arguing for its presence as either study motivation or data interpretation, yet failing to show convincing evidence through results. Taken together with results of the current analysis, this lack of evidence leads us to reconsider the assumption that pathological co-contraction is a primary factor contributing to impaired gait post-stroke. The following discussion enumerates the details leading to this conclusion.

Early studies assessing EMG during gait post-stroke carefully described the authors’ observations while presenting mostly qualitative evaluations and proposing future avenues of research. Knutsson and Richards are often credited for the suggestion that co-contraction could be a strategy employed in post-stroke gait, but they carefully acknowledged that the heterogeneity present within their sample limited their ability to draw distinct conclusions (22). Another group classified whole-leg muscle activation patterns, classifying less than half of their sample in the chronic phase of recovery as expressing excessive co-contraction (5). There is heterogeneity in virtually all motor outcomes measured following stroke, and that heterogeneity impacts the ability to assess treatment efficacy (44). Equally important, no strong evidence has emerged in favor of co-contraction as either the predominant strategy or one of a few common strategies employed by these individuals. Moreover, the evidence provides no indication that mitigating co-contraction is a productive treatment target. Working under the assumption that co-contraction indices provide useful information, therefore, limits our ability to appropriately quantify motor impairment within this population.

Heterogeneity of responses among individuals post-stroke leads many groups to seek a single metric, or a simple collection of metrics, which can parse these individual differences. Here, we employed a two-factor approach by combining EMG metrics with measures of motor or gait impairment. Although our data reveal some significant correlations, these are not sufficiently strong to support an argument for a causal link between co-contraction and impaired biomechanical function. Lower extremity coordination during gait requires much more than the ankle muscles; however, the vital importance of the ankle plantarflexors affords an ideal test-bed for assessing the relevance of lower extremity co-contraction post-stroke. Yet, no clear patterns emerged. It is worth noting that four of the individuals within this sample regularly wore some form of ankle brace. The extent to which bracing impacted the EMG patterns differentially from the stroke itself is not known. We compared these participants against those who did not wear a brace and found no apparent differences. We acknowledge this is a limitation within our sample that may be worth exploring in the future. It is also important to note that EMG assesses the final common pathway (45), and these peripheral signals cannot be used to draw specific conclusions about central nervous system function. Although we expect surface EMG signals to provide information about motor coordination, co-contraction may not be sufficiently robust to explain central nervous system dysfunction.

Changing the co-contraction metric offered variable results. Contrary to our initial expectations, fixing the biomechanical roles of the muscles was only marginally more informative than the existing CI, which was not designed to account for abnormal muscle activation patterns. Importantly, the group-level results were the same; neither metric revealed differences between healthy individuals and individuals post-stroke. On the group level, even the WC metric performed similarly to the two co-contraction indices. However, thorough evaluation of the relationship of the individual numeric values to the EMG profiles provides insight that the WC metric does not perform well during gait. From production of extreme outliers to equivalent numeric values in obviously different EMG patterns (Figure 5), we can conclusively state that the WC metric is not useful for assessment of co-contraction in multi-segmental lower extremity tasks.

Normalization also provided varying results, creating a need for careful consideration of normalization procedures before, during, and after EMG assessment. Perhaps the most interesting result from the normalization analysis is the congruency of the M-max and non-normalized EMG co-contraction indices. Choosing not to normalize EMG can be advantageous because it avoids data transformation beyond recognition. Our findings support arguments presented by Lamontagne regarding EMG metrics in stroke (6). While EMG normalization is not strictly necessary in this context because calculating a ratio from normalized data effectively normalizes the data twice, most studies in the literature employ some type of normalization prior to calculating a co-contraction index (46–48). In our sample, maximum value normalization suggested a different picture of relative muscle activation than either M-max normalization or no normalization.

Co-contraction does not appear to be a universal characteristic of impaired gait post-stroke. We do not mean to suggest that excessive co-contraction does not occur in some individuals; however, the pattern does not occur sufficiently often enough to be considered a prominent compensatory strategy following stroke. Ours is not the first study to find a lack of strong evidence for the presence of excessive co-contraction following stroke. In both the upper and lower extremities, the current evidence suggests that reaching and walking deficits, respectively, are more likely to result from agonist activation impairment than co-contraction (49, 50). It is possible that co-contraction represents too narrow of a concept to represent and characterize neuromotor pathology post-stroke. Accordingly, it may be time to reconsider how we frame the problem. Aberrant EMG is descriptively characterized by timing and amplitude deficits, and the ability to organize responses to the biomechanical constraints of the task and environment (51). In this broader context, approaches other than a co-contraction metric may better capture the relevant deficits. Some recent efforts that have yet to be robustly evaluated in stroke include: Ricamato and Hidler’s EMG metric that incorporates both timing and amplitude components (52); muscle synergy analysis (53, 54); and EMG-driven biomechanical modeling that effectively accounts for subject-specific neuromuscular constraints on dynamic outcomes (55). Our intent is not to prescribe any one of these methods to adequately quantify neurophysiologic impairments with EMG. Rather, we would argue that these and other alternatives be explored in an effort to learn more about the causal mechanisms of gait impairment following stroke.

Beyond an exercise in signal processing and EMG data analysis, our results provide an opportunity for discussion regarding the neural implications of co-contraction following stroke. Heterogeneity among individuals also presents a challenge for understanding behavior after stroke, especially in terms of muscle activation patterns. Our data illustrate that, even when assessed with a variety of metrics, co-contraction does not emerge as the strong indicator of neuromotor pathology the literature has conditioned us to expect. Instead, we must look to other EMG quantification methods that can provide greater insights regarding causal mechanisms of gait impairment.

## Ethics Statement

All procedures were approved by the University of Florida Health Science Center Institutional Review Board (IRB-01) and all participants gave written informed consent prior to enrollment. Testing was conducted in accordance with the Declaration of Helsinki.

## Author Contributions

CB, HH, and CP conceived and designed experiments. CB and HH contributed analysis tools, prepared figures, and analyzed data. CB and VL performed experiments. CB, HH, VL, and CP interpreted results, revised manuscript, approved final version of manuscript, and agreed to be accountable for all aspects of the work. CB drafted manuscript.

## Conflict of Interest Statement

The authors declare that the research was conducted in the absence of any commercial or financial relationships that could be construed as a potential conflict of interest.
